# Thirty Years with HIV Infection—Nonprogression Is Still Puzzling: Lessons to Be Learned from Controllers and Long-Term Nonprogressors

**DOI:** 10.1155/2012/161584

**Published:** 2012-05-27

**Authors:** Julie C. Gaardbo, Hans J. Hartling, Jan Gerstoft, Susanne D. Nielsen

**Affiliations:** Department of Infectious Diseases, Rigshospitalet, Copenhagen University Hospital, Copenhagen 2100, Denmark

## Abstract

In the early days of the HIV epidemic, it was observed that a minority of the infected patients did not progress to AIDS or death and maintained stable CD4+ cell counts. As the technique for measuring viral load became available it was evident that some of these nonprogressors in addition to preserved CD4+ cell counts had very low or even undetectable viral replication. They were therefore termed controllers, while those with viral replication were termed long-term nonprogressors (LTNPs). Genetics and virology play a role in nonprogression, but does not provide a full explanation. Therefore, host differences in the immunological response have been proposed. Moreover, the immunological response can be divided into an immune homeostasis resistant to HIV and an immune response leading to viral control. Thus, non-progression in LTNP and controllers may be due to different immunological mechanisms. Understanding the lack of disease progression and the different interactions between HIV and the immune system could ideally teach us how to develop a functional cure for HIV infection. Here we review immunological features of controllers and LTNP, highlighting differences and clinical implications.

## 1. Introduction

Prior to the introduction of combination antiretroviral therapy (cART) it was observed that a minority of the individuals infected with Human Immunodeficiency Virus type 1 (HIV-1, from now on referred to as HIV) did not progress to Acquired Immunodeficiency Syndrome (AIDS) or death. This minority maintained normal CD4+ cell counts in the absence of treatment for several years—in some cases for more than two decades (reviewed in [1]) and therefore the terminology Long-Term Nonprogressors (LTNP) was proposed. When the technique for measuring the viral load was introduced it became evident that some of these patients, who did not clinically progress, had low or even nondetectable viral replication. This phenomenon leads to the additive definition of the non progressor-phenotype referred to as controllers due to their ability to control viral replication in the absence of cART. Today, non-progressors are a collective name for controllers and LTNP who are clinically similar. Except for certain demands for the duration of the infection in LTNP, they are only to be differentiated according to control or not of viral replication, respectively. Understanding the mechanism for the lack of disease progression in controllers and LNTP could ideally teach us how to develop a functional cure for HIV infection, and for this reason these subpopulations of HIV-infected patients have gained immense interest.

Non-progressors are described to differ from progressors in genetics, virology, and immunology. Genetically, certain factors seem to predispose to non-progressions. Thus, it has been shown that female gender, the presence of CCR5-delta32 polymorphism, and HLA genes, in particular the HLA B57 allele, are overrepresented among non-progressors ([2–9], reviewed in [10]). Virologically, a number of studies have indeed shown that some non-progressors are infected with less virulent strains of HIV resulting in a more benign infection [11–14]. However, there are now several lines of evidence that the majority of non-progressors are infected with replicant-competent virus [15–17]. One study has shown that CD4+ cells from controllers are less susceptible to HIV compared to CD4+ cells from progressors and healthy controls [18], while another study showed that CD4+ cells from controllers were as susceptible or even more susceptible to HIV entry and productive infection [19].

Thus, genes and viral factors play a role in non-progression, but these components do not provide a thorough explanation. For this reason the immune system is to be considered a key element in non-progression. This is supported by the recent demonstrations of better control of hepatitis C virus (HCV) in HCV-infected controllers compared to HCV-infected progressors [20, 21]. The immunological response to HIV infection can be divided into (1) an immune homeostasis resistant to HIV in LTNP and (2) the immune-mediated control of the virus in controllers. However, despite these two possible interactions between HIV and the immune system both resulting in preserved CD4+ cell counts, few studies have compared immune homeostasis in LTNP and controllers. The scope of this paper is to describe immunology in non-progressing HIV infection and to propose involved immunological mechanisms in LTNP and controllers.

## 2. Definitions of Nonprogressors

It is well-established that LTNP and controllers are different subpopulations [22–25], supporting the idea that different immunological mechanisms are responsible for the preserved CD4+ cell counts. LTNP and controllers are described as rare populations comprising few percentages of all HIV-infected individuals, and with little overlap between them [22–27], although the definition of the populations suffers from lack of consensus in terminology and inclusion criteria, impeding the comparison of findings.

Controllers can be further divided into elite controllers (EC) and viremic controllers (VCs), most commonly with HIV RNA <50 copies/mL and 50–2000 copies/mL, respectively, although variations with higher levels are found as well ([25], reviewed in[27, 28]). This is in particular a problem because LTNP can thereby be categorized as VC due to their often relatively low viremia. In addition, a central problem in defining the non-progressor phenotype is a complete lack of inclusion of the viral load, thereby including LTNP who fulfill controller-criteria with low or undetectable viral loads.

Patients need not necessarily be infected for a long period of time in order to be categorized as controllers. Thus, two measurements of a low viral load during one year are sometimes used as sufficient, whereas others demand duration of infection for several years. In contrast, LTNPs due to the nature of the case definition have a long duration of infection, most commonly a minimum of 7–10 years. Both groups present with a CD4+ cell count within the normal range (350–1600 cells/*μ*L) [22, 23, 25, 29]. Due to the low prevalence of these non-progressors, it is tempting to relax the inclusion criteria. However, clinical outcomes seem to improve with the stringency of criteria, and it has been demonstrated that the clinical outcome for patients infected for 7 versus 10 years and with stable CD4+ cell counts is different. Thus, a better survival among LTNP defined by 10 years of stable infection versus 7 years is reported [25], suggesting that even 7 years of stable infection do not distinguish properly between true LTNP and progressors.

In this paper, unless anything else mentioned, the term “non-progressors” is used as a collective name for controllers and LTNP, while the term “controllers” is used for VC and EC. The LTNP term is only used when studied patients had substantial viremia above 2000 copies/mL and had been infected for a minimum of 7 years. Although 7 years may not be enough to exclusively distinguish LTNP from progressors, 7 years are chosen as most studies have used this definition. The duration of infection in controllers is not included in our definitions as we consider viral control at any time point to be a sufficient determinant for controller status (Table 1).

## 3. Immunology in Non-Progressors

The CD4+ cell count in a given patient at any time is the result of production, destruction, and traffic between blood and lymphatic tissue, and when the destruction exceeds the production the CD4+ cell count decreases. Thus, LTNP and controllers may have differences in production, destruction, or distribution of CD4+ cells compared to progressors in order to maintain a normal CD4+ cell count.

## 4. Production of Cells

### 4.1. Bone Marrow and Progenitor Cells

T cells mature in the thymus, but they originate from hematopoietic progenitor cells (HPCs) in the bone marrow (BM). Thus, a functional BM is crucial for thymopoiesis. In the hope of developing a cure for HIV infection HPC has been given greater attention, even more so after the report on eradication of HIV by transplantation of CCR5-deficient HPC in the so-called Berlin patient [30]. HIV influences BM and HPC, and impaired hematopoiesis in HIV infection is well documented [31–34]. Furthermore, several studies have shown that some HPCs express the HIV receptors CD4, CXCR4, and CCR5 making them potentially susceptible to HIV infection (reviewed in [35]). HIV infection of HPC has recently been suggested [36], although the complexities of purifying and maintaining HPC in culture make it difficult to determine if these HPCs are actually infected, as signs of infection may be due to contamination with other cell types or maturation of HPC to monocytes during in vitro culture. However, HIV proteins seem sufficient to disturb the haematopoiesis [37]. Nevertheless, in addition to T cells, natural killer cells and B cells, including naïve B cells, seem to be depleted during HIV infection [38]. HIV-associated lymphopenia may therefore be explained by more upstream elements of lymphocyte development than reduced thymic output.

Circulating HPCs have been found to decrease with disease progression and to be associated with CD4+ cell count [39], supporting the idea of BM and HPC as being essential in preservation of CD4+ cell count and suggesting preserved haematopoiesis in non-progressors. The haematopoiesis has only been assessed in a single study of elite controllers (Table 2). This study included progressing as well as non-progressing EC. Interestingly, the progressing EC showed signs of exhausted lymphopoiesis compared the non-progressing EC measured as CD34+ cells and lymphoid-HPC [39]. This is supportive of a sufficient haematopoiesis as a contributing factor to non-progression in controllers. Also, it indicates that the viral replication itself is not the only reason for disease progression.

### 4.2. Thymus and Naive Cells

The CD4+ cell count is maintained by proliferation of already existing CD4+ cells or by de novo production in the thymus. Earlier, it was believed that the thymus was only active in childhood and replaced by fatty tissue with increasing age. It is now evident that the thymus can also be active in adulthood, particularly during circumstances with lymphopenia, as is the case with HIV infection [40, 41]. Often thymic function is assessed as T*-*cell receptor excision circles (TRECs) or as the naive CD4+ cell count. TRECs are stable circular DNA fragments that are excised during the formation of TCR in the maturing T cell in the thymus, and TRECs are not replicated during cell division. Thus, the more immature CD4+ cells the higher the TREC content. A large thymus on CT scans has been associated with a higher CD4+ TREC frequency in HIV-infected patients [42]. Thus, TRECs and naive cells are all reasonable indirect measurements of thymic size and output.

HIV leads to a disruption in the number and function of naïve CD4+ cells in blood as well as in lymphoid tissue [43–45]. To our knowledge, no studies of naïve cells have discriminated between controllers and LTNP. In non-progressors, similar and lower numbers of naïve CD4+ cells have been found compared to progressors [8, 46, 47], suggesting that the level of naïve CD4+ cells itself is not associated with non-progression (Table 2). In contrast, increased expression of the naïve marker CCR7, higher levels of central memory cells with preserved ability to secrete interleukin 2 (IL-2), and a much higher thymic output as defined by TRECs in EC compared to progressors have been reported [46, 48], supporting preserved thymic function in non-progressors. The contribution of a well-functioning thymopoiesis to non-progression is further supported by the findings of strong correlations between TRECs and non-progression in SIV-infected rhesus macaques [49]. Also, normal levels of memory cells and preserved IL-2 secretion capacities have been shown in non-progressing SIV-infected rhesus macaques compared to progressors [50]. For this reason, it seems plausible that the thymic function is better in non-progressors compared to progressors, improving their ability to maintain a normal CD4+ cell count. However, these findings only explain the preserved CD4+ cell count in non-progressors, not the viral control in controllers. In fact, the high thymic output may indirectly be a consequence of and not a reason for the low viral replication, since lower viral replication does not lead to the exhaustion of lymphopoiesis normally seen in progressors [39]. Thus, it would be of great interest to compare the thymic output in LTNP and progressors since both populations have viral replication. It is tempting to assume that one of the main differences between these progressors and LTNP is an extraordinary capacity to produce cells. This is supported by findings of higher levels of naïve cells in a study of slow-compared to fast progressors based on the slope of their CD4+ cell loss, although this did not reach statistical significance [51]. Likewise, a study of children with LTNP status displayed higher levels of naïve cells compared to progressors and controls [52].

Thymic output is dramatically reduced with age, and the naïve cells are increasingly generated from peripheral proliferation (reviewed in [53]). Proliferation of cells leads to a lower TREC count, and therefore naïve cells in older individuals have lower TREC counts [54, 55]. Thus, the reported loss of the non-progressor status in some individuals may be due to increasing age and thereby decreased thymic output, because the thymus is no longer able to meet the demands of a high production of cells. This is supported by the findings of an immune system in HIV-infected patients which is comparable to much older healthy individuals [39]. Also, high age is a predictor of poor immune reconstitution after initiation of cART [56].

### 4.3. IL7

Production of CD4+cells is influenced by Interleukin 7 (IL-7). IL-7 is crucial in the T-cell homeostasis, and the IL-7 responsiveness is determined largely by the presence or absence of the IL-7 receptor (IL-7R), which is present on most mature T cells [57]. A negative correlation between IL-7 and CD4+ cell count is described. Consequently, HIV-infected progressors have high levels of IL-7 and reduced levels of IL-7R compared to healthy controls [58, 59], consistent with the need for increased production of CD4+ cells and a down-regulation of the receptor due to high plasma levels. Thus, controllers would be expected to display a pattern of IL-7/IL-7R closer to healthy controls than to progressors. This is supported by findings of lower levels of IL7 in controllers compared to controllers who lost their controller status [60] and by findings of lower levels of IL-7R in progressors compared to non-progressors [47]. In contrast, it would make sense that LTNPs display a pattern of IL-7/IL-7R more like progressors than controllers, because the need for CD4+ cell replenishment would be expected to depend on the level of viral replication—infection and cell—turnover, which is supported by findings from our own lab (unpublished data) These results are compatible with a hypothesis of low viral replication leading to a reduced number of new CD4+ cells becoming infected. Thereby, the level of IL-7 does not increase, and the IL-7R expression stays high. However, further studies are warranted to clarify this.

## 5. Destruction of Cells

### 5.1. Immune Activation, Senescence, and Apoptosis

Immune activation (IA) is a necessary and normal acute response upon infection with any pathogen, as an effort to avoid infection. However, in HIV-infected individuals it is well established that chronic IA is linked to and predictive of disease progression, and IA has an additive or stronger prognostic value than does CD4+ cell count or viral load alone [61–68]. The influence of IA on disease progression can be partly explained by elevated levels of senescent and apoptotic cells as a consequence of IA, thereby leading to increased loss of CD4+ cells.

Elevated markers of activation are to be found in most cell compartments, but especially expression of the surface markers CD38 and HLA-DR on CD8+ cells have proven to be predictive of disease progression [64–68]. Thus, IA is a central player in disease progression, as illustrated by the development of pneumocystic pneumonia in rats solely as a consequence of IA [69]. In light of this, it is obvious to assume that IA in non-progressors is different from progressors and partly explains lack of progression. IA is one of the more well-examined features in non-progressors and has in several studies been found to be lower in EC as well as VC compared to progressors [70–74]. In support of the significance of low IA on lack of disease progression a study of EC revealed that the individuals with the highest IA presented with the lowest CD4+ cell counts [73] (Table 2). Also, the natural hosts of simian immunodeficiency virus (SIV), sooty mangabeys and African green monkeys, who do not progress despite a high viral load (and thus can be seen as a simian pendant to LTNP), do not show any signs of increased IA or T*-*cell turnover [75, 76]. IA is inadequately examined in LTNP. One study did not find any differences in IA between EC and LTNP, while they were both different from progressors [77], while another study did not find any differences between the three groups [78]. Interestingly, this latter study, one of the only studies to have compared controllers and LTNP, also evaluated the phenotypic and functional properties of CD56/CD16 natural killer (NK) cells, a major component of the innate immune system. Cytolytic activity against autologous CD4+ cells was found to be abrogated after treatment with an antibody to NKp44L, the cellular ligand of the natural cytotoxicity receptor NKp44, which is specifically induced on CD4+ T cells during HIV-1 infection, in LTNP and HIV progressors. In contrast, in HIV controllers and healthy donors, NKp44L expression on CD4+ cells and autologous NK lysis were both poorly detected [78]. This is strongly supportive of LTNP and controllers as being immunologically different.

Another component of the innate immune system that may be involved in non-progression is the plasmacytoid Dendritic Cells (pDCs). PDCs have been suggested as inducers of IA and CD4+ cell apoptosis as they recognize HIV single-stranded RNA (ssRNA) via Toll-like receptors (TLR) resulting in interferon (INF) *α* production [79–82]. Furthermore, polymorphisms in TLR7 and the interferon regulator 7 of INF*α* may influence disease progression and the ability of pDCs to produce INF*α* [83, 84]. In controllers, the number and function of pDCs are reported to be preserved [85]. In addition, pDCs from rhesus macaques produce large amounts of INF*α* when stimulated with SIV or HIV, while the natural hosts, sooty mangabeys, seem to have lower levels of INF*α* [86]. Altogether, this suggests pDCs to induce IA, and they may therefore be involved in non-progression of HIV infection.

IA is accompanied by apoptosis and immunological senescence, and HIV-infected patients present with elevated levels of both features [87, 88]. Senescent cells in EC and progressors have been examined in a single study, and comparable levels were found [8]. In contrast, in a study of non-progressors with unknown viral load the level of apoptosis was found to be similar to healthy controls and lower than in progressors [89], and others have reported lower levels of apoptosis in VC compared to progressors [90], both supporting the idea of a lower turnover as contributing to non-progression. However, low turnover could also simply reflect a lower IA. Contrary, another study found elevated levels of apoptotic cells in non-progressors compared to progressors [91], implying that a high turnover is beneficial. This finding makes sense, if we assume that activated cells are eliminated by apoptosis. Then an increasing proportion of apoptotic cells eliminate the harmful IA, thereby diminishing disease progression. Either way, evidence is lacking, and whether a high turnover of cells is contributing to non-progression is still to be determined.

Thus, it seems reasonable to assume that the level of IA is a determinant for how fast the turnover of T cells is, thereby relating IA to exhaustion (Table 2). Indeed, it has been proposed that IA leads to CD4+ cell depletion because it erodes the naïve T-cell pool [92]. Still, the reason for the strong predictive value of IA in HIV infection is uncertain, but low IA found in controllers suggests that IA forms an important role in lack of progression.

## 6. Immune Regulation: Pro- and Anti-Inflammatory Cells

The understanding of the immune system is constantly changing as a consequence of rapidly expanding knowledge. Recently, the discovery of T-cell subsets with pro- and anti-inflammatory properties has altered our view on immunology. Regulatory T cells (Tregs) are anti-inflammatory T cells, while Th17 cells have proinflammatory properties. Tregs are crucial in sustaining tolerance to self-antigens [93, 94] and suppressing T-cell activation resulting in down-regulation of immune activation, including reduction in anti-tumor immunity, graft rejection, and graft *versus* host disease ([95], reviewed in [96]). Finally, the role of Tregs in chronic viral infections, including HIV, has gained considerable interest due to their immunosuppressive capabilities. Thus, in theory, Tregs can downregulate the chronic IA seen in HIV infection making Tregs a key element in the understanding of the interaction between the host immune system and HIV (reviewed in [97]). For this reason, Tregs have been suggested as downregulators of the unbeneficial unspecific IA in HIV-infection, expecting high levels of Tregs as being an advantage. However, levels of Tregs in HIV-infected, untreated, progressing patients have been shown to be elevated compared to healthy controls in a number of studies ([98–100] reviewed in [101]). This suggests that high levels of Tregs are actually harmful, possibly because they downregulate beneficial HIV-specific responses. In support of this, the level of Tregs in controllers has been reported to be lower compared to progressors, and closer to healthy controls, although conflicting results have been reported as well [70, 77, 102–106]. Furthermore, it has been shown that the suppressive activity of Tregs in EC is preserved, while it was found to be disrupted in progressors [105]. Finally, Tregs have been suggested to increase with age (reviewed in [107]), possibly contributing to the reported loss of non-progression in some individuals. All together this is supportive of a significant influence of Tregs on non-progression.

However, like most other pieces in the puzzle of understanding the interaction of HIV and the immune system the Treg element has proven to be more complex than expected. Thus, Tregs are closely related to IL-17-producing Th17 cells. Tregs and Th17 cells share a reciprocal maturation pathway and function together in opposing ways to control the inflammatory response upon infection. While Tregs inhibit autoimmunity, Th17 cells have been shown to play a critical role in the induction of autoimmune tissue injury and immune responses [108]. Th17 cells have been shown to be rapidly depleted during acute SIV infection cells, and a disturbed balance of Th17 cells and Tregs has been suggested to be associated with subsequent high IA and disease progression ([109, 110], reviewed in [101, 111]). In controllers, a maintained balance between Tregs and Th17 cells is reported [102] (reviewed in [112]), highlighting the significance of a well-regulated balance between Tregs and Th17 cells. Th17 cells and Tregs have primarily been examined in controllers. However, one study of a group of non-progressors, where most participants met the LTNP criteria, have found elevated levels of Th17 cells compared to progressors [113]. Thus, a high level of Th17 cells may contribute to lack of progression in controllers as well as in LTNP. However, HIV leads to redistribution of CD4+ cells between blood and lymphatic tissue (LT). Thus, it has been demonstrated that HIV binds to resting CD4+ cells and upregulates L-selectin causing the cells to home from the blood into lymph nodes (LNs) at enhanced rates [114, 115]. This has led to the homing theory, offering an explanation for the loss of CD4+ cells due to cells leaving the blood and entering the LT (reviewed in [116]). Indeed, accumulation of Tregs has been found in secondary lymphatic tissue (SLT) compared to peripheral blood in untreated HIV-infected patients [117, 118], indicating that the conclusion drawn from the reported findings of Tregs in peripheral blood is to be considered with caution.

## 7. Secondary Lymphatic Tissue and Microbial Translocation

CD4+ cell depletion occurs in the blood as well as in the SLT of LN and gut-associated lymphatic tissue (GALT) where the majority of the CD4+ cells reside. During primary HIV infection a vast number of cells are depleted, reaching a loss of more than 50% in LN as chronic infection is established [119, 120]. It has been proposed that HIV damages the structures in the LT, that help sustain the normal CD4+ cell population replacing the functional space with collagen. Thus, the greater the amount of the collagen deposition, the lower the CD4+ cell count and the smaller the number of naive CD4+ cells [121]. Also, LN biopsies from HIV- and SIV-infected individuals show breakdown of the lymph node architecture and evidence of apoptosis [122]. In contrast, a preserved lymph-node architecture was reported in the history of HIV in non-progressors compared to progressors, indicating that progressors host a preserved SLT [123]. GALT is the main defence against infectious microorganisms in the gastrointestinal (GI) tract and consists largely of T cells. Importantly, the main part of Th17 cells reside in the GALT [124]. Th17 cells are important for the integrity of the gut mucosal barrier by stimulating epithelial proliferation and inducing a proinflammatory environment by recruiting neutrophils to fight microorganisms. Upon acute HIV infection follows a significant depletion of CD4+ cells in the GALT [125, 126]. The depletion is linked to a damage of the mucosal barrier that may be due to an imbalance of Th17 cells as the massive depletion of CD4+ cells during acute HIV and SIV infection in particular includes Th17 cells [125, 126]. The damage to the mucosal barrier results in microbial translocation (MT)—a continuing leak of microbial remnants from the GI tract that enters the systemic circulation. These microbial products lead to immune activation [111, 127, 128], thereby contributing to HIV progression. The data on mucosal integrity and the influence of MT on immune activation in non-progressors are limited. One study using a rhesus macaque model has shown that spontaneous restoration of mucosal CD4+ cells upon acute SIV infection is predictive of non-progression [129]. Furthermore, EC and VC present with similar preserved numbers of CD4+ cells in rectal biopsies comparable to HIV-negative individuals, while the number in progressors is reported to be diminished [130, 131]. However, the level of lipopolysaccharide (LPS) used as a marker of MT is comparable in controllers and progressors and elevated compared to HIV-negative individuals [73], indicating that low chronic immune activation in non-progressors might have effect in the long-term despite the appearance of the relatively intact mucosal barrier. Thus, present data indicate that non-progressors are distinct from progressors in several aspects of the integrity of the mucosal barrier and MT, suggesting an important mechanism for the capability of non-progressors to control immune activation and HIV infection. However, to determine the causal relationship between MT and control of HIV infection prospective studies are needed.

## 8. HIV-Specific Immune Responses

HIV-specific CD8+ and CD4+ cells and neutralizing antibodies are considered an important albeit most often insufficient element in suppressing viral replication (reviewed in [132, 133]). Some of the first studies were made of HIV-infected patients with primary infection. Here it was shown that the level of HIV-specific CD8+ cells paralleled the efficiency of control of primary viremia. Also, patients who mounted strong gp160-specific CD8+ cell responses showed rapid reduction of acute plasma viremia, while viremia in patients with low virus specific CD8+ cell activity was poorly controlled [134]. Another study showed that an absent HIV-specific CD8+ cell response during primary HIV infection was associated with prolonged symptoms, persistent viremia, and low CD4+ T-cell count [135]. Furthermore, it has been shown that in vivo depletion of CD8+ cells eliminates the ability to contain SIV replication [136]. For this reason an HIV-specific CD8+ cell immune response is widely accepted as a contributor to control of viral replication and lack of progression in non-progressors, and this has been evaluated in a number of studies. Thus, it has been shown that non-progressors are able to maintain an established CD8+ cell precursor pool and present with a consistent highly functional HIV-specific response, while this ability is lost in progressors [137, 138]. Also, the capacity of virus-specific CD8+ cells to proliferate in response to stimulation with HIV antigens is reported to be preserved only in non-progressors [139]. This is in agreement with findings from a prospective study of an increase in polyfunctionality in HIV-specific CD8+ cell responses from EC, and a decrease in progressors over time [140], and with findings of a stronger and broader cytokine and chemokine response following HIV-specific stimulation of PBMC from EC compared to progressors [70, 77]. In addition, it has been reported that the inhibitory immunoregulatory receptor CTLA-4 is selectively upregulated in HIV-specific CD4+ cells in progressors compared to non-progressors. CTLA-4 expression was also found to be positive associated with disease progression and negatively associated with the capacity of CD4+ cells to produce IL-2 in response to viral antigen [141]. Furthermore, it has been shown that in non-progressors HIV-specific CD8+ T cells efficiently eliminate primary autologous HIV-infected CD4+ cells [142]. Additionally, it seems of importance if the HIV-specific cells are activated or not, as it has been shown that ECs possess lower levels of activated HIV-specific CD8+ cells and of recently divided HIV-specific CD4+ cells than progressors [70]. Based on these data an ideal HIV vaccine would induce strong HIV-specific immune responses and minimize HIV-specific immune activation. Another goal of vaccine development is induction of antibodies that neutralize a broad range of HIV isolates. Although antibodies can be elicited by HIV infection, those that are broadly neutralizing are undetectable in most individuals (reviewed in [143]). The level and the breadth of neutralizing antibodies are reported to correlate to viral load [144, 145], and the same or lower levels of antibodies are reported in controllers compared to progressors [144, 146]. Furthermore, one study showed that no single anti-HIV antibody specificity was a clear correlate of immunity in controllers [146]. This is consistent with neutralizing antibodies as poor contributors to non-progression. Contrary, antibodies directed against autologous Env variants are reported to be present in non-progressors [147], and efficient elicitation of de novo neutralizing antibodies has been shown in SIV controllers [148].

Taken together these findings unanimously indicate that a virus-specific response by CD8+ cells is a contributing factor to non-progression, while the influence of virus-specific CD4+ cells and neutralizing antibodies is more unclear. Interestingly, similar preserved HIV-specific T-cell responses have been demonstrated in a study of controllers and LTNPs while responses were blunted in progressors [149]. This indicates that HIV-specific responses are crucial in sustaining non-progression, regardless the viral replication, consequently playing a role in non-progression in controllers as well as in LTNP. However, these beneficial HIV-specific responses might be a part of the explanation of why HIV is not being eradicated, even in controllers. Thus, it has recently been shown that high HIV-specific responses are associated with high levels of cell-associated HIV DNA levels in controllers [150].

## 9. Eradication, Latency, and Reservoirs

Despite effective cART complete eradication of HIV seems unlikely, and complete eradication of HIV is so far only obtained once in the Berlin-patient [30]. In general, low-level HIV replication continues despite cART. This is in part due to the capability of HIV to conceal itself and persist in cellular reservoirs. Furthermore, a major impediment to the eradication of HIV is latently infected resting CD4+ cells that are characterized by proviral DNA integration into the host genome; particulary memory CD4+ cells have proven to be a major cellular reservoir for HIV [151]. The major anatomical site for HIV reservoir is SLT including GALT [152–154]. Thus, viral reservoirs are considered a major obstacle to eradicate HIV and considered to be the reason for rebound viraemia during cART interruptions. Notably, the concept of a functional cure has emerged where lifelong control of viral replication is obtained and disease progression is avoided although provirus is detectable. This might be illustrated by the viral control found EC.

Few studies have examined the capability of non-progressors to eradicate or reduce HIV reservoirs and latency. However, low proviral DNA in PBMC in EC compared to patients on cART has been demonstrated [155, 156]. One study even reported differences in the level of proviral loads in EC compared to VC, implying that even low viral replication is of importance [17]. Furthermore, autologous viral replication ex vivo was detected in only 2 out of 14 EC compared to 9 out of 10 VCs, suggesting ECs to have a diminished viral reservoir in peripheral CD4+ cell compartment [17]. Furthermore, impaired viral replication in the early phase of HIV seems to be predictive for VC [157]. The anatomical reservoir in GALT has been examined in a small study revealing lower levels of HIV DNA in rectal tissue in LTNP compared to progressors [158]. However, an SIV macaque model demonstrated that colon mucosa and associated lymph nodes are a major SIV reservoir even in controllers [159].

Conclusively, non-progressors and especially EC seem to have diminished cellular reservoirs, whereas the size of the anatomical viral reservoirs in SLT is uncertain. However, the described traffic of CD4+ cells between plasma and SLT and the reduced microbial translocation indicate that non-progressors harbor a lower viral reservoir in SLT than do progressors. Understanding how ECs control and reduce cellular reservoir might provide the basis to elucidate the needs for a functional cure.

## 10. Clinical Implications

A common feature in non-progressors is preserved immunology. However, reports of loss of the non-progressor status with declining CD4+ cell counts in LTNP have been observed, and these patients may eventually require cART. Likewise, a loss of viral control in controllers is reported. Interestingly, progression and AIDS events in controllers despite low or undetectable viral loads events are reported as well [2, 8, 22, 29, 39, 48, 73, 160]. As a result of this, cART has been suggested to controllers.

In EC viral replication cannot be measured in commercial assays in EC. However, using ultrasensitive assays HIV RNA can be detected in the majority of these patients [161, 162], and it has been shown that loss of CD4+ cells is more common among ECs with low level viremia [162]. Furthermore, low-level viremia is reported to be associated with measurable T cell dysfunction in EC [163]. Finally, is has been shown that blips are associated with a nonfavourable clinical outcome [26]. Taken together, this indicates that even very low levels of viremia have clinical implications and are involved in disease progression. This is supported by findings of higher levels of immune activation in controllers compared to patients on cART [73, 164]. Moreover, it seems plausible that cART would normalize immunological parameters in controllers. This is confirmed by findings of declining immune activation in EC and increasing CD4+ cell counts in EC as well as EC as due to cART initiation [165, 166]. Thus, cART may be considered in progressing controllers despite undetectable viral replication.

## 11. Conclusion and Future Directions

Rare groups of HIV-infected patients that do not progress to AIDS or death have been known since the beginning of the HIV epidemic. Some of these non-progressors control viral replication, that is, the controllers, while LTNP, have ongoing viral replication. So far, it is not clear why these patients do not progress, but immunological mechanisms have been suggested. The immunological response to HIV infection can be divided into an immune homeostasis resistant to HIV and an immune response leading to viral control. This paper has focused on immunology in non-progressors. We suggest that two different mechanisms are responsible for preserved CD4+ cell counts in controllers and LTNP.

In summary, data unambiguously show that controllers are immunologically different from progressors in production, destruction, and regulation of cells. Thus, controllers have a preserved CD4+ cell production with a bone marrow function, a lymphopoiesis, a thymic output, and an IL-7/IL7-R balance resembling HIV-negative individuals. Furthermore, controllers have lower destruction of CD4+ cells as evidenced by lower microbial translocation, immune activation, and apoptosis. Likewise, the balance between Tregs and Th17 cells is less disturbed and HIV reservoirs seem to be lower compared to progressors. However, non-progression and preserved CD4+ cells counts in controllers may not be entirely surprising since they are characterized by viral control and thus to be compared with HIV-infected patients on treatment. Thus, preserved immune homeostasis may be a reflection of rather than a reason for the viral control. In contrast, high level of HIV-specific immune response in controllers is probably a contributing factor to non-progression in controllers.

The really intriguing question is how non-progression occurs in LTNP where continuous viral replication is evident and ought to result in destruction of CD4+ cells. Unfortunately, the literature of LTNP is limited, often because they are included in study populations of controllers as viral load is not included in the definitions. An extraordinary ability to produce cells or a lower rate of destruction is expected in these patients according to the preservation of normal CD4+ cell counts. However, increased rate of production has not been shown so far, and the finding of similar levels of IL-7 between LTNP and progressors suggests that a different CD4+ production is not the explanation for non-progression in LTNP. Likewise, evidence of reduced turnover of cells in LTNP has not been found, and in general LTNPs appear to have an immune system very much like progressors. However, findings of elevated levels of Th17 cells have been reported, suggesting that the immune regulation by pro- and anti-inflammatory cells is different in LTNP compared to progressors. Indeed, it would be interesting to further evaluate immunological parameters, includingTh17 cells, Tregs, and microbial translocation in LTNP, ideally in prospective studies in order to clarify cause and effect. Also, it would be of interest to elucidate immunological parameters in former LTNP who have lost their non-progressor status.

Finally, due to distinct immunologically profiles in LTNP and controllers, we suggest that a clear distinction between patients with and without viral replication is made in future studies in order to improve the possibility to understand the different mechanisms for non-progression in these fascinating patients.

## Figures and Tables

**Table 1:** Definitions of non-progressors.

	EC	VC	LTNP
CD4+ cell count (cells/*μ*L)	>350	>350	>350
Viral load (copies/mL)	<50	50–200	>2000
Duration of infection (years)	*	*	>7
cART	No	No	No

*Duration of infection is not used in the definition of controllers in this review.

**Table 2:** Immunological distinctions between progressors and non-progressors.

	Controller	LTNP	Progressor
Production of T cells	Thymic output preserved	Thymus output?	Thymic output ↓
	Haematopoiesis preserved	Haematopoiesis?	Haematopoiesis exhausted

IL-7/IL-7R	IL-7 Normal	IL-7 ↑	IL-7 ↑
	IL-7R Normal	IL-7R ↓	IL-7R ↓

Destruction of T cells	Immune activation ↑	Immune activation ↑↑	Immune activation ↑↑↑
	Turnover/apoptosis ↑	Turnover/apoptosis?	Turnover/apoptosis ↑↑
	Microbial translocation ↑	? Microbial translocation	↑↑↑Microbial translocation

Secondary lymphoid tissue	Preserved architecture	?	Damaged architecture

Pro- and anti-inflammatory cells	↓Tregs	? Tregs	↑↑Tregs
	↑Th17	↑Th17	↓Th17

HIV-specific immune response	Strong	?	Blunted

Viral reservoir	Low	?	High

↑/↓: Indicates slightly, ↑↑/↓↓: moderate, ↑↑↑/↓↓↓: highly different from HIV-negative individuals.

LTNP: long-term non-progressors, Tregs: regulatory T cells.

?: Indicates unknown.

## References

[1] Mikhail M, Wang B, Saksena NK (2003). Mechanisms involved in non-progressive HIV disease. *AIDS Reviews*.

[2] Madec Y, Boufassa F, Porter K, Meyer L (2005). Spontaneous control of viral load and CD4 cell count progression among HIV-1 seroconverters. *AIDS*.

[3] Dean M, Carrington M, Winkler C (1996). Genetic restriction of HIV-1 infection and progression to AIDS by a deletion allele of the CKR5 structural gene. Hemophilia Growth and Development Study, Multicenter AIDS Cohort Study, Multicenter Hemophilia Cohort Study, San Francisco City Cohort, ALIVE Study. *Science*.

[4] Eugen-Olsen J, Iversen AKN, Garred P (1997). Heterozygosity for a deletion in the CKR-5 gene leads to prolonged AIDS-free survival and slower CD4 T-cell decline in a cohort of HIV-seropositive individuals. *AIDS*.

[5] Klein MR, Van Der Burg SH, Hovenkamp E (1998). Characterization of HLA-B57-restricted human immunodeficiency virus type 1 Gag- and RT-specific cytotoxic T lymphocyte responses. *Journal of General Virology*.

[6] Piacentini L, Biasin M, Fenizia C, Clerici M (2009). Genetic correlates of protection against HIV infection: the ally within. *Journal of Internal Medicine*.

[7] Fellay J, Shianna KV, Ge D (2007). A whole-genome association study of major determinants for host control of HIV-1. *Science*.

[8] Ruiz-Mateos E, Ferrando-Martinez S, Machmach K (2010). High levels of CD57^+^CD28^−^T-cells, low T-cell proliferation and preferential expansion of terminally differentiated CD4^+^ T-cells in HIV-Elite controllers. *Current HIV Research*.

[9] Pereyra F, Jia X, McLaren PJ, Telenti A, De Bakker PIW, Walker BD (2010). The major genetic determinants of HIV-1 control affect HLA class I peptide presentation. *Science*.

[10] Poropatich K, Sullivan DJ (2011). Human immunodeficiency virus type 1 long-term non-progressors: the viral, genetic and immunological basis for disease non-progression. *Journal of General Virology*.

[11] Kirchhoff F, Greenough TC, Brettler DB, Sullivan JL, Desrosiers RC (1995). Brief report: absence of intact nef sequences in a long-term survivor with nonprogressive HIV-1 infection. *New England Journal of Medicine*.

[12] Mariani R, Kirchhoff F, Greenough TC, Sullivan JL, Desrosiers RC, Skowronski J (1996). High frequency of defective nef alleles in a long-term survivor with nonprogressive human immunodeficiency virus type 1 infection. *Journal of Virology*.

[13] Iversen AKN, Shpaer EG, Rodrigo AG (1995). Persistence of attenuated rev genes in a human immunodeficiency virus type 1-infected asymptomatic individual. *Journal of Virology*.

[14] Deacon NJ, Tsykin A, Solomon A (1995). Genomic structure of an attenuated quasi species of HIV-1 from a blood transfusion donor and recipients. *Science*.

[15] Blankson JN, Bailey JR, Thayil S (2007). Isolation and characterization of replication-competent human immunodeficiency virus type 1 from a subset of elite suppressors. *Journal of Virology*.

[16] Bailey JR, O’Connell K, Yang HC (2008). Transmission of human immunodeficiency virus type 1 from a patient who developed AIDS to an elite suppressor. *Journal of Virology*.

[17] Julg B, Pereyra F, Buzón MJ (2010). Infrequent recovery of HIV from but robust exogenous infection of activated CD4^+^ T Cells in HIV Elite Controllers. *Clinical Infectious Diseases*.

[18] Chen H, Li C, Huang J (2011). CD4^+^ T cells from elite controllers resist HIV-1 infection by selective upregulation of p21. *Journal of Clinical Investigation*.

[19] Rabi SA, O’Connell KA, Nikolaeva D (2011). Unstimulated primary CD4^+^ T cells from HIV-1-positive elite suppressors are fully susceptible to HIV-1 entry and productive infection. *Journal of Virology*.

[20] Ruiz-Mateos E, MacHmach K, Romero-Sanchez MC (2011). Hepatitis C virus replication in caucasian HIV controllers. *Journal of Viral Hepatitis*.

[21] Sajadi MM, Shakeri N, Talwani R, Redfield RR (2010). Hepatitis C infection in HIV-1 natural viral suppressors. *AIDS*.

[22] Grabar S, Selinger-Leneman H, Abgrall S, Pialoux G, Weiss L, Costagliola D (2009). Prevalence and comparative characteristics of long-term nonprogressors and HIV controller patients in the French Hospital Database on HIV. *AIDS*.

[23] Petrucci A, Dorrucci M, Alliegro MB (1997). How many HIV-infected individuals may be defined as long-term nonprogressors? A report from the Italian Seroconversion Study. Italian Seroconversion Study Group (ISS). *Journal of Acquired Immune Deficiency Syndromes & Human Retrovirology*.

[24] Lambotte O, Boufassa F, Madec Y (2005). HIV controllers: a homogeneous group of HIV-1-infected patients with spontaneous control of viral replication. *Clinical Infectious Diseases*.

[25] Okulicz JF, Marconi VC, Landrum ML (2009). Clinical outcomes of elite controllers, viremic controllers, and long-term nonprogressors in the US department of defense HIV natural history study. *Journal of Infectious Diseases*.

[26] Boufassa F, Saez-Cirion A, Lechenadec J (2011). CD4 dynamics over a 15 year-period among HIV controllers enrolled in the ANRS French Observatory. *PLoS ONE*.

[27] Hunt PW (2009). Natural control of HIV-1 replication and long-term nonprogression: overlapping but distinct phenotypes. *Journal of Infectious Diseases*.

[28] Saag M, Deeks SG (2010). How do HIV elite controllers do what they do?. *Clinical Infectious Diseases*.

[29] Rodés B, Toro C, Paxinos E (2004). Differences in disease progression in a cohort of long-term non-progressors after more than 16 years of HIV-1 infection. *AIDS*.

[30] Hütter G, Ganepola S (2011). Eradication of HIV by transplantation of CCR5-deficient hematopoietic stem cells. *The Scientific World Journal*.

[31] Moses A, Nelson J, Bagby GC (1998). The influence of human immunodeficiency virus-1 on hematopoiesis. *Blood*.

[32] Marandin A, Katz A, Oksenhendler E (1996). Loss of primitive hematopoietic progenitors in patients with human immunodeficiency virus infection. *Blood*.

[33] Nielsen SD, Afzelius P, Dam-Larsen S (1998). Effect of granulocyte colony-stimulating factor (G-CSF) in human immunodeficiency virus-infected patients: increase in numbers of naive CD4 cells and CD34 cells makes G-CSF a candidate for use in gene therapy or to support antiretroviral therapy. *Journal of Infectious Diseases*.

[34] Nielsen SD, Clark DR, Hutchings M (1999). Treatment with granulocyte colony-stimulating factor decreases the capacity of hematopoietic progenitor cells for generation of lymphocytes in human immunodeficiency virus-infected persons. *Journal of Infectious Diseases*.

[35] Alexaki A, Wigdahl B (2008). HIV-1 infection of bone marrow hematopoietic progenitor cells and their role in trafficking and viral dissemination. *PLoS Pathogens*.

[36] Carter CC, Onafuwa-Nuga A, McNamara LA (2010). HIV-1 infects multipotent progenitor cells causing cell death and establishing latent cellular reservoirs. *Nature Medicine*.

[37] Nielsen SD, Jeppesen DL, Kolte L (2001). Impaired progenitor cell function in HIV-negative infants of HIV-positive mothers results in decreased thymic output and low CD4 counts. *Blood*.

[38] Moir S, Ho J, Malaspina A (2008). Evidence for HIV-associated B cell exhaustion in a dysfunctional memory B cell compartment in HIV-infected viremic individuals. *Journal of Experimental Medicine*.

[39] Sauce D, Larsen M, Fastenackels S (2011). HIV disease progression despite suppression of viral replication is associated with exhaustion of lymphopoiesis. *Blood*.

[40] Douek DC, Vescio RA, Betts MR (2000). Assessment of thymic output in adults after haematopoietic stem-cell transplantation and prediction of T-cell reconstitution. *The Lancet*.

[41] Haynes BF, Markert ML, Sempowski GD, Patel DD, Hale LP (2000). The role of the thymus in immune reconstitution in aging, bone marrow transplantation, and HIV-1 infection. *Annual Review of Immunology*.

[42] Kolte L, Dreves AM, Ersbøll AK (2002). Association between larger thymic size and higher thymic output in human immunodeficiency virus-infected patients receiving highly active antiretroviral therapy. *Journal of Infectious Diseases*.

[43] Hazenberg MD, Otto SA, Stuart JWTC (2000). Increased cell division but not thymic dysfunction rapidly affects the T-cell receptor excision circle content of the naive T cell population in HIV-1 infection. *Nature Medicine*.

[44] Douek DC, McFarland RD, Keiser PH (1998). Changes in thymic function with age and during the treatment of HIV infection. *Nature*.

[45] Autran B, Carcelain G, Li TS (1997). Positive effects of combined antiretroviral therapy on CD4^+^ T cell homeostasis and function in advanced HIV disease. *Science*.

[46] Potter SJ, Lacabaratz C, Lambotte O (2007). Preserved central memory and activated effector memory CD4^+^ T-cell subsets in human immunodeficiency virus controllers: an ANRS EP36 study. *Journal of Virology*.

[47] Marchetti G, Riva A, Cesari M (2009). HIV-infected long-term nonprogressors display a unique correlative pattern between the interleukin-7/interleukin-7 receptor circuit and T-cell homeostasis. *HIV Medicine*.

[48] Westrop SJ, Qazi NA, Pido-Lopez J (2009). Transient nature of long-term nonprogression and broad virus-specific proliferative T-cell responses with sustained thymic output in HIV-1 controllers. *PLoS ONE*.

[49] Fang RHT, Khatissian E, Monceaux V (2005). Disease progression in macaques with low SIV replication levels: on the relevance of TREC counts. *AIDS*.

[50] He H, Nehete PN, Nehete B (2011). Functional impairment of central memory CD4 T cells is a potential early prognostic marker for changing viral load in SHIV-infected rhesus macaques. *PLoS ONE*.

[51] Goicoechea M, Smith D, May S, Mathews C, Spina C (2009). Prevalence and T-cell phenotype of slow HIV disease progressors with robust HIV replication. *Journal of Acquired Immune Deficiency Syndromes*.

[52] Resino S, Correa R, Bellon JM, Munoz-Fernandez MA (2003). Preserved immune system in long-term asymptomatic vertically HIV-1 infected children. *Clinical & Experimental Immunology*.

[53] Khoury G, Rajasuriar R, Cameron PU, Lewin SR (2011). The role of naive T-cells in HIV-1 pathogenesis: an emerging key playerc. *Clinical Immunology*.

[54] Kimmig S, Przybylski GK, Schmidt CA (2002). Two subsets of naive T helper cells with distinct T cell receptor excision circle content in human adult peripheral blood. *Journal of Experimental Medicine*.

[55] Junge S, Kloeckener-Gruissem B, Zufferey R (2007). Correlation between recent thymic emigrants and CD31^+^ (PECAM-1) CD4^+^ T cells in normal individuals during aging and in lymphopenic children. *European Journal of Immunology*.

[56] Engsig FN, Gerstoft J, Kronborg G (2010). Long-term mortality in hiv patients virally suppressed for more than three years with incomplete CD4 recovery: a cohort study. *BMC Infectious Diseases*.

[57] Park LS, Friend DJ, Schmierer AE, Dower SK, Namen AE (1990). Murine interleukin 7 (IL-7) receptor. Characterization on an IL-7-dependent cell line. *Journal of Experimental Medicine*.

[58] Llano A, Barretina J, Gutiérrez A (2001). Interleukin-7 in plasma correlates with CD4 T-cell depletion and may be associated with emergence of syncytium-inducing variants in human immunodeficiency virus type 1-positive individuals. *Journal of Virology*.

[59] Rethi B, Fluur C, Atlas A (2005). Loss of IL-7R*α* is associated with CD4 T-cell depletion, high interleukin-7 levels and CD28 down-regulation in HIV infected patients. *AIDS*.

[60] Fluur C, Rethi B, Thang PH (2007). Relationship between serum IL-7 concentrations and lymphopenia upon different levels of HIV immune control. *AIDS*.

[61] Giorgi JV, Hultin LE, McKeating JA (1999). Shorter survival in advanced human immunodeficiency virus type 1 infection is more closely associated with T lymphocyte activation than with plasma virus burden or virus chemokine coreceptor usage. *Journal of Infectious Diseases*.

[62] Sousa AE, Carneiro J, Meier-Schellersheim M, Grossman Z, Victorino RMM (2002). CD4 T cell depletion is linked directly to immune activation in the pathogenesis of HIV-1 and HIV-2 but only indirectly to the viral load. *Journal of Immunology*.

[63] Grossman Z, Meier-Schellersheim M, Sousa AE, Victorino RMM, Paul WE (2002). CD4^+^ T-cell depletion in HIV infection: are we closer to understanding the cause?. *Nature Medicine*.

[64] Hazenberg MD, Otto SA, Van Benthem BHB (2003). Persistent immune activation in HIV-1 infection is associated with progression to AIDS. *AIDS*.

[65] Liu Z, Cumberland WG, Hultin LE, Kaplan AH, Detels R, Giorgi JV (1998). CD8^+^ T-lymphocyte activation in HIV-1 disease reflects an aspect of pathogenesis distinct from viral burden and immunodeficiency. *Journal of Acquired Immune Deficiency Syndromes & Human Retrovirology*.

[66] Liu Z, Cumberland WG, Hultin LE, Prince HE, Detels R, Giorgi JV (1997). Elevated CD38 antigen expression on CD8^+^ T cells is a stronger marker for the risk of chronic HIV disease progression to AIDS and death in the Multicenter AIDS Cohort Study than CD4^+^ cell count, soluble immune activation markers, or combinations of HLA-DR and CD38 expression. *Journal of Acquired Immune Deficiency Syndromes & Human Retrovirology*.

[67] Liu Z, Hultin LE, Cumberland WG (1996). Elevated relative fluorescence intensity of CD38 antigen expression on CD8^+^ T cells is a marker of poor prognosis in HIV infection: results of 6 years of follow-up. *Cytometry*.

[68] Giorgi JV, Liu Z, Hultin LE, Cumberland WG, Hennessey K, Detels R (1993). Elevated levels of CD38^+^ CD8^+^ T cells in HIV infection add to the prognostic value of low CD4^+^ T cell levels: results of 6 years of follow-up. The Los Angeles Center, Multicenter AIDS Cohort Study. *Journal of Acquired Immune Deficiency Syndromes*.

[69] Pesanti EL (1982). Effects of bacterial pneumonitis on development of pneumocystosis in rats. *American Review of Respiratory Disease*.

[70] Owen RE, Heitman JW, Hirschkorn DF (2010). HIV+ elite controllers have low HIV-specific T-cell activation yet maintain strong, polyfunctional T-cell responses. *AIDS*.

[71] Kamya P, Tsoukas CM, Boulet S (2011). T cell Activation does not drive CD4 decline in longitudinally followed HIV-infected Elite Controllers. *AIDS Research and Therapy*.

[72] Bello G, Velasco-de-Castro CA, Bongertz V (2009). Immune activation and antibody responses in non-progressing elite controller individuals infected with HIV-1. *Journal of Medical Virology*.

[73] Hunt PW, Brenchley J, Sinclair E (2008). Relationship between T cell activation and CD4^+^ T cell count in HIV-seropositive individuals with undetectable plasma HIV RNA levels in the absence of therapy. *Journal of Infectious Diseases*.

[74] Carbone J, Gil J, Benito JM, Fernandez-Cruz E (2003). Decreased expression of activation markers on CD4 T lymphocytes of HIV-infected long-term non-progressors. *AIDS*.

[75] Broussard SR, Staprans SI, White R, Whitehead EM, Feinberg MB, Allan JS (2001). Simian immunodeficiency virus replicates to high levels in naturally infected African green monkeys without inducing immunologic or neurologic disease. *Journal of Virology*.

[76] Kaur A, Grant RM, Means RE, Mcclure H, Feinberg M, Johnson RP (1998). Diverse host responses and outcomes following simian immunodeficiency virus SIVmac239 infection in sooty mangabeys and rhesus macaques. *Journal of Virology*.

[77] Whittall T, Peters B, Rahman D, Kingsley CI, Vaughan R, Lehner T (2011). Immunogenic and tolerogenic signatures in human immunodeficiency virus (HIV)-infected controllers compared with progressors and a conversion strategy of virus control. *Clinical & Experimental Immunology*.

[78] Vieillard V, Fausther-Bovendo H, Samri A, Debre P (2010). Specific phenotypic and functional features of natural killer cells from HIV-infected long-term nonprogressors and HIV controllers. *Journal of Acquired Immune Deficiency Syndromes*.

[79] Heil F, Hemmi H, Hochrein H (2004). Species-specific recognition of single-stranded RNA via Till-like receptor 7 and 8. *Science*.

[80] Chang JJ, Altfeld M (2009). TLR-mediated immune activation in HIV. *Blood*.

[81] Meier A, Chang JJ, Chan ES (2009). Sex differences in the Toll-like receptor-mediated response of plasmacytoid dendritic cells to HIV-1. *Nature Medicine*.

[82] Stary G, Klein I, Kohlhofer S (2009). Plasmacytoid dendritic cells express TRAIL and induce CD4^+^ T-cell apoptosis in HIV-1 viremic patients. *Blood*.

[83] Oh DY, Baumann K, Hamouda O (2009). A frequent functional toll-like receptor 7 polymorphism is associated with accelerated HIV-1 disease progression. *AIDS*.

[84] Chang J, Lindsay RJ, Kulkarni S, Lifson JD, Carrington M, Altfeld M (2011). Polymorphisms in interferon regulatory factor 7 reduce interferon-*α* responses of plasmacytoid dendritic cells to HIV-1. *AIDS*.

[85] Machmach K, Leal M, Gras C (2012). Plasmacytoid dendritic cells reduce HIV production in elite controllers. *Journal of Virology*.

[86] Mandl JN, Barry AP, Vanderford TH (2008). Divergent TLR7 and TLR9 signaling and type I interferon production distinguish pathogenic and nonpathogenic AIDS virus infections. *Nature Medicine*.

[87] Meyaard L, Otto SA, Jonker RR, Mijnster MJ, Keet RPM, Miedema F (1992). Programmed death of T cells in HIV-1 infection. *Science*.

[88] Palmer BE, Blyveis N, Fontenot AP, Wilson CC (2005). Functional and phenotypic characterization of CD57^+^CD4^+^ T cells and their association with HIV-1-induced T cell dysfunction. *Journal of Immunology*.

[89] Franceschi C, Franceschini MG, Boschini A (1997). Phenotypic characteristics and tendency to apoptosis of peripheral blood mononuclear cells from HIV+ long term non progressors. *Cell Death and Differentiation*.

[90] Schweneker M, Favre D, Martin JN, Deeks SG, McCune JM (2008). HIV-induced changes in T cell signaling pathways. *Journal of Immunology*.

[91] Zanussi S, Simonelli C, D’Andrea M (1996). CD8^+^ lymphocyte phenotype and cytokine production in long-term non-progressor and in progressor patients with HIV-1 infection. *Clinical and Experimental Immunology*.

[92] Hazenberg MD, Hamann D, Schuitemaker H, Miedema F (2000). T cell depletion in HIV-1 infection: how CD4^+^ T cells go out of stock. *Nature Immunology*.

[93] Read S, Mauze S, Asseman C, Bean A, Coffman R, Powrie F (1998). CD38^+^ CD45RB^low^ CD4^+^ T cells: a population of T cells with immune regulatory activities in vitro. *European Journal of Immunology*.

[94] Baecher-Allan C, Brown JA, Freeman GJ, Hafler DA (2001). CD4^+^CD25^high^ regulatory cells in human peripheral blood. *Journal of Immunology*.

[95] Yoshizawa A, Ito A, Li Y (2005). The roles of CD25^+^CD4^+^ regulatory T cells in operational tolerance after living donor liver transplantation. *Transplantation Proceedings*.

[96] Sakaguchi S (2005). Naturally arising Foxp3-expressing CD25^+^CD4^+^ regulatory T cells in immunological tolerance to self and non-self. *Nature Immunology*.

[97] Bernardes SS, Borges IK, Lima JE (2010). Involvement of regulatory T cells in HIV immunopathogenesis. *Current HIV Research*.

[98] Gaardbo JC, Nielsen SD, Vedel SJ (2008). Regulatory T cells in human immunodeficiency virus-infected patients are elevated and independent of immunological and virological status, as well as initiation of highly active anti-retroviral therapy. *Clinical and Experimental Immunology*.

[99] Weiss L, Donkova-Petrini V, Caccavelli L, Balbo M, Carbonneil C, Levy Y (2004). Human immunodeficiency virus-driven expansion of CD4^+^CD25^+^ regulatory T cells, which suppress HIV-specific CD4 T-cell responses in HIV-infected patients. *Blood*.

[100] Lim A, Tan D, Price P (2007). Proportions of circulating T cells with a regulatory cell phenotype increase with HIV-associated immune activation and remain high on antiretroviral therapy. *AIDS*.

[101] Kanwar B, Favre D, McCune JM (2010). Th17 and regulatory T cells: implications for AIDS pathogenesis. *Current Opinion in HIV and AIDS*.

[102] Brandt L, Benfield T, Mens H (2011). Low level of regulatory T cells and maintenance of balance between regulatory T cells and TH17 cells in HIV-1-infected elite controllers. *Journal of Acquired Immune Deficiency Syndromes*.

[103] Jiao Y, Fu J, Xing S (2009). The decrease of regulatory T cells correlates with excessive activation and apoptosis of CD8^+^ T cells in HIV-1-infected typical progressors, but not in long-term non-progressors. *Immunology*.

[104] Li L, Liu Y, Bao Z (2011). Analysis of CD4^+^CD25^+^Foxp3^+^ regulatory T cells in HIV-exposed seronegative persons and HIV-infected persons with different disease progressions. *Viral Immunology*.

[105] Chase AJ, Yang HC, Zhang H, Blankson JN, Siliciano RF (2008). Preservation of FoxP3^+^ regulatory T cells in the peripheral blood of human immunodeficiency virus type 1-infected elite suppressors correlates with low CD4^+^ T-cell activation. *Journal of Virology*.

[106] Hunt PW, Landay AL, Sinclair E (2011). A low T regulatory cell response may contribute to both viral control and generalized immune activation in HIV controllers. *PLoS ONE*.

[107] Wang L, Xie Y, Zhu LJ, Chang TT, Mao YQ, Li J (2010). An association between immunosenescence and CD4^+^CD25^+^ regulatory T cells: a systematic review. *Biomedical and Environmental Sciences*.

[108] Bettelli E, Carrier Y, Gao W (2006). Reciprocal developmental pathways for the generation of pathogenic effector TH17 and regulatory T cells. *Nature*.

[109] Favre D, Lederer S, Kanwar B (2009). Critical loss of the balance between Th17 and T regulatory cell populations in pathogenic SIV infection. *PLoS Pathogens*.

[110] Favre D, Mold J, Hunt PW (2010). Tryptophan catabolism by indoleamine 2, 3-dioxygenase 1 alters the balance of TH17 to regulatory T cells in HIV disease. *Science Translational Medicine*.

[111] Hunt PW (2010). Th17, gut, and HIV: therapeutic implications. *Current Opinion in HIV and AIDS*.

[112] Hartigan-O’Connor DJ, Hirao LA, McCune JM, Dandekar S (2011). Th17 cells and regulatory T cells in elite control over HIV and SIV. *Current Opinion in HIV and AIDS*.

[113] Salgado M, Rallon NI, Rodes B, Lopez M, Soriano V, Benito JM (2011). Long-term non-progressors display a greater number of Th17 cells than HIV-infected typical progressors. *Clinical Immunology*.

[114] Wang L, Robb CW, Cloyd MW (1997). HIV induces homing of resting T lymphocytes to lymph nodes. *Virology*.

[115] Wang L, Chen JJY, Gelman BB, Konig R, Cloyd MW (1999). A novel mechanism of CD4 lymphocyte depletion involves effects of HIV on resting lymphocytes: induction of lymph node homing and apoptosis upon secondary signaling through homing receptors. *Journal of Immunology*.

[116] Cloyd MW, Chen JJY, Adeqboyega P, Wang L (2001). How does HIV cause depletion of CD4 lymphocytes? A mechanism involving virus signaling through its cellular receptors. *Current Molecular Medicine*.

[117] Epple HJ, Loddenkemper C, Kunkel D (2006). Mucosal but not peripheral FOXP3^+^ regulatory T cells are highly increased in untreated HIV infection and normalize after suppressive HAART. *Blood*.

[118] Shaw JM, Hunt PW, Critchfield JW (2011). Increased frequency of regulatory T cells accompanies increased immune activation in rectal mucosae of HIV-positive noncontrollers. *Journal of Virology*.

[119] Mattapallil JJ, Douek DC, Hill B, Nishimura Y, Martin M, Roederer M (2005). Massive infection and loss of memory CD4^+^ T cells in multiple tissues during acute SIV infection. *Nature*.

[120] Guadalupe M, Reay E, Sankaran S (2003). Severe CD4^+^ T-cell depletion in gut lymphoid tissue during primary human immunodeficiency virus type 1 infection and substantial delay in restoration following highly active antiretroviral therapy. *Journal of Virology*.

[121] Schacker TW, Nguyen PL, Beilman GJ (2002). Collagen deposition in HIV-1 infected lymphatic tissues and T cell homeostasis. *Journal of Clinical Investigation*.

[122] Finkel TH, Tudor-Williams G, Banda NK (1995). Apoptosis occurs predominantly in bystander cells and not in productively infected cells of HIV- and SIV-infected lymph nodes. *Nature Medicine*.

[123] Pantaleo G, Menzo S, Vaccarezza M (1995). Studies in subjects with long-term nonprogressive human immunodeficiency virus infection. *New England Journal of Medicine*.

[124] Brenchley JM, Douek DC (2008). The mucosal barrier and immune activation in HIV pathogenesis. *Current Opinion in HIV and AIDS*.

[125] Brenchley JM, Schacker TW, Ruff LE (2004). CD4^+^ T cell depletion during all stages of HIV disease occurs predominantly in the gastrointestinal tract. *Journal of Experimental Medicine*.

[126] Mehandru S, Poles MA, Tenner-Racz K (2004). Primary HIV-1 infection is associated with preferential depletion of CD4^+^ T lymphocytes from effector sites in the gastrointestinal tract. *Journal of Experimental Medicine*.

[127] Merlini E, Bai F, Bellistrì GM, Tincati C, d’Arminio Monforte A, Marchetti G (2011). Evidence for polymicrobic flora translocating in peripheral blood of HIV-infected patients with poor immune response to antiretroviral therapy. *PLoS ONE*.

[128] Brenchley JM, Price DA, Schacker TW (2006). Microbial translocation is a cause of systemic immune activation in chronic HIV infection. *Nature Medicine*.

[129] Ling B, Veazey RS, Hart M (2007). Early restoration of mucosal CD4 memory CCR5 T cells in the gut of SIV-infected rhesus predicts long term non-progression. *AIDS*.

[130] Ferre AL, Hunt PW, Critchfield JW (2009). Mucosal immune responses to HIV-1 in elite controllers: a potential correlate of immune control. *Blood*.

[131] Ferre AL, Hunt PW, McConnell DH (2010). HIV controllers with HLA-DRB1^∗^13 and HLA-DQB1^∗^06 alleles have strong, polyfunctional mucosal CD4^+^ T-cell responses. *Journal of Virology*.

[132] Migueles SA, Tilton JC, Connors M (2004). Advances in understanding immunologic control of HIV infection. *Current HIV/AIDS Reports*.

[133] Porichis F, Kaufmann DE (2011). HIV-specific CD4 T cells and immune control of viral replication. *Current Opinion in HIV and AIDS*.

[134] Borrow P, Lewicki H, Hahn BH, Shaw GM, Oldstone MBA (1994). Virus-specific CD8^+^ cytotoxic T-lymphocyte activity associated with control of viremia in primary human immunodeficiency virus type 1 infection. *Journal of Virology*.

[135] Koup RA, Safrit JT, Cao Y (1994). Temporal association of cellular immune responses with the initial control of viremia in primary human immunodeficiency virus type 1 syndrome. *Journal of Virology*.

[136] Letvin NL, Schmitz JE, Jordan HL (1999). Cytotoxic T lymphocytes specific for the simian immunodeficiency virus. *Immunological Reviews*.

[137] Pontesilli O, Klein MR, Kerkhof-Garde SR (1998). Longitudinal analysis of human immunodeficiency virus type 1-specific cytotoxic T lymphocyte responses: a predominant gag-specific response is associated with nonprogressive infection. *Journal of Infectious Diseases*.

[138] Betts MR, Nason MC, West SM (2006). HIV nonprogressors preferentially maintain highly functional HIV-specific CD8^+^ T cells. *Blood*.

[139] Jagannathan P, Osborne CM, Royce C (2009). Comparisons of CD8^+^ T cells specific for human immunodeficiency virus, hepatitis C virus, and cytomegalovirus reveal differences in frequency, immunodominance, phenotype, and interleukin-2 responsiveness. *Journal of Virology*.

[140] Peris-Pertusa A, Lopez M, Rallon NI, Restrepo C, Soriano V, Benito JM (2010). Evolution of the functional profile of HIV-specific CD8^+^ T cells in patients with different progression of HIV infection over 4 years. *Journal of Acquired Immune Deficiency Syndromes*.

[141] Kaufmann DE, Kavanagh DG, Pereyra F (2007). Upregulation of CTLA-4 by HIV-specific CD4^+^ T cells correlates with disease progression and defines a reversible immune dysfunction. *Nature Immunology*.

[142] Migueles SA, Osborne CM, Royce C (2008). Lytic granule loading of CD8^+^ T cells is required for HIV-infected cell elimination associated with immune control. *Immunity*.

[143] Verkoczy L, Kelsoe G, Moody MA, Haynes BF (2011). Role of immune mechanisms in induction of HIV-1 broadly neutralizing antibodies. *Current Opinion in Immunology*.

[144] Doria-Rose NA, Klein RM, Daniels MG (2010). Breadth of human immunodeficiency virus-specific neutralizing activity in sera: clustering analysis and association with clinical variables. *Journal of Virology*.

[145] Sajadi MM, Guan Y, DeVico AL (2011). Correlation between circulating HIV-1 RNA and broad HIV-1 neutralizing antibody activity. *Journal of Acquired Immune Deficiency Syndromes*.

[146] Lambotte O, Ferrari G, Moog C (2009). Heterogeneous neutralizing antibody and antibody-dependent cell cytotoxicity responses in HIV-1 elite controllers. *AIDS*.

[147] Mahalanabis M, Jayaraman P, Miura T (2009). Continuous viral escape and selection by autologous neutralizing antibodies in drug-naïve human immunodeficiency virus controllers. *Journal of Virology*.

[148] Yamamoto H, Kawada M, Tsukamoto T (2007). Vaccine-based, long-term, stable control of simian/human immunodeficiency virus 89.6PD replication in rhesus macaques. *Journal of General Virology*.

[149] Choudhary SK, Vrisekoop N, Jansen CA (2007). Low immune activation despite high levels of pathogenic human immunodeficiency virus type 1 results in long-term asymptomatic disease. *Journal of Virology*.

[150] Hunt PW, Hatano H, Sinclair E (2011). HIV-specific CD4^+^ T cells may contribute to viral persistence in HIV controllers. *Clinical Infectious Diseases*.

[151] Chomont N, El-Far M, Ancuta P (2009). HIV reservoir size and persistence are driven by T cell survival and homeostatic proliferation. *Nature Medicine*.

[152] Chun TW, Carruth L, Finzi D (1997). Quantification of latent tissue reservoirs and total body viral load in HIV-1 infection. *Nature*.

[153] Chun TW, Nickle DC, Justement JS (2008). Persistence of HIV in gut-associated lymphoid tissue despite long-term antiretroviral therapy. *Journal of Infectious Diseases*.

[154] Brenchley JM, Price DA, Schacker TW (2006). Microbial translocation is a cause of systemic immune activation in chronic HIV infection. *Nature Medicine*.

[155] Graf EH, Mexas AM, Yu JJ (2011). Elite suppressors harbor low levels of integrated HIV DNA and high levels of 2-LTR circular HIV DNA compared to HIV+ patients on and off HAART. *PLoS Pathogens*.

[156] Sáez-Cirión A, Hamimi C, Bergamaschi A (2011). Restriction of HIV-1 replication in macrophages and CD4^+^ T cells from HIV controllers. *Blood*.

[157] Miura T, Brumme ZL, Brockman MA (2010). Impaired replication capacity of acute/early viruses in persons who become HIV controllers. *Journal of Virology*.

[158] Avettand-Fenoel V, Prazuck T, Hocqueloux L (2008). HIV-DNA in rectal cells is well correlated with HIV-DNA in blood in different groups of patients, including long-term non-progressors. *AIDS*.

[159] Ling B, Mohan M, Lackner AA (2010). The large intestine as a major reservoir for simian immunodeficiency virus in macaques with long-term, nonprogressing infection. *Journal of Infectious Diseases*.

[160] Lefrère JJ, Morand-Joubert L, Mariotti M (1997). Even individuals considered as long-term nonprogressors show biological signs of progression after 10 years of human immunodeficiency virus infection. *Blood*.

[161] Hatano H, Delwart EL, Norris PJ (2009). Evidence for persistent low-level viremia in individuals who control human immunodeficiency virus in the absence of antiretroviral therapy. *Journal of Virology*.

[162] Pereyra F, Palmer S, Miura T (2009). Persistent low-level viremia in HIV-1 elite controllers and relationship to immunologic parameters. *Journal of Infectious Diseases*.

[163] Pereyra F, Addo MM, Kaufmann DE (2008). Genetic and immunologic heterogeneity among persons who control HIV infection in the absence of therapy. *Journal of Infectious Diseases*.

[164] López M, Soriano V, Peris-Pertusa A, Rallón N, Restrepo C, Benito JM (2011). Elite controllers display higher activation on central memory CD8 T cells than HIV patients successfully on HAART. *AIDS Research and Human Retroviruses*.

[165] Okulicz JF, Grandits GA, Weintrob AC (2010). CD4 T cell count reconstitution in HIV controllers after highly active antiretroviral therapy. *Clinical Infectious Diseases*.

[166] Sedaghat AR, Rastegar DA, OConnell KA, Dinoso JB, Wilke CO, Blankson JN (2009). T cell dynamics and the response to HAART in a cohort of HIV-1-infected elite suppressors. *Clinical Infectious Diseases*.

